# Smallholders’ uneven capacities to adapt to climate change amid Africa’s ‘green revolution’: Case study of Rwanda’s crop intensification program

**DOI:** 10.1016/j.worlddev.2018.11.022

**Published:** 2019-04

**Authors:** Nathan Clay, Brian King

**Affiliations:** a School of Geography and the Environment, University of Oxford, Oxford Martin School, 34 Broad St., Oxford OX1 3BD, UK; b Department of Geography, The Pennsylvania State University, 312A Walker Building, University Park, PA 16802, USA

**Keywords:** Adaptation, Agricultural intensification, Climate change, Institutions, Livelihoods, Vulnerability

## Abstract

Development programs and policies can influence smallholder producers’ abilities to adapt to climate change. However, gaps remain in understanding how households’ adaptive capacities can become uneven. This paper investigates how development transitions—such as the recent adoption of ‘green revolution’ agricultural policies throughout sub-Saharan Africa—intersect with cross-scale social-environmental processes to unevenly shape smallholders’ adaptive capacities and adaptation pathways. Drawing on quantitative and qualitative material from a multi-season study in Rwanda, we investigate smallholder adaptation processes amid a suite of rural development interventions. Our study finds that adaptive capacities arise differentially across livelihood groups in the context of evolving environmental, social, and political economic processes. We show how social institutions play key roles in shaping differential adaptation pathways by enabling and/or constraining opportunities for smallholders to adapt livelihood and land use strategies. Specifically, Rwanda’s Crop Intensification Program enables some wealthier households to adapt livelihoods by generating income through commercial agriculture. At the same time, deactivation of local risk management institutions has diminished climate risk management options for most households. To build and employ alternate livelihood practices such as commercial agriculture and planting woodlots for charcoal production, smallholders must negotiate new institutions, a prerequisite for which is access to capitals (land, labor, and nonfarm income). Those without entitlements to these are pulled deeper into poverty with each successive climatic shock. This illustrates that adaptive capacity is not a static, quantifiable entity that exists in households. We argue that reconceptualizing adaptive capacity as a dynamic, social-environmental process that emerges in places can help clarify complex linkages among development policies, livelihoods, and adaptation pathways. To ensure more equitable and climate-resilient agricultural development, we stress the need to reformulate policies with careful attention to how power structures and entrenched social inequalities can lead to smallholders’ uneven capacities to adapt to climate change.

## Highlights

• Rwanda’s Crop Intensification Program has inhibited smallholder mechanisms of climate risk management.
• To mitigate climatic risks, smallholders must employ resources to adapt land use strategies.
• Wealthier households have enrolled in new institutions that are increasingly essential to building adaptive capacity.
• Lacking resources and robust livelihood portfolios, poorer households struggle to cope with and adapt to climatic change.

## 1 Introduction

Looming over smallholder producers in sub-Saharan Africa (SSA) are constellations of climate change and ambitious development interventions that aim to enhance agricultural productivity as a way to end hunger and stimulate economic growth. Climate change models predict increased temperatures and erratic rainfall patterns that will likely challenge current modes of production and exacerbate food insecurity across the region (Niang et al., 2014, Muller et al., 2011, Schmidhuber and Tubiello, 2007). A recent report by the Intergovernmental Panel on Climate Change (IPCC) notes that the 1.5 degree Celcius increase in mean global temperature expected by 2030 will severely impact rural livelihoods and human health in SSA (Hoegh-Guldberg et al., 2018). Meanwhile, development efforts in SSA have re-centered on smallholder agriculture as an engine of economic growth. Large-scale agricultural intensification schemes have proliferated across SSA over the past decade in effort to close crop ‘yield gaps’ (Lobell, Cassman, & Field, 2009) and catalyze a ‘Green Revolution for Africa’ (United Nations, 2004, World Bank, 2007, Diao et al., 2010). Within this context of rapid social-environmental change, smallholders (those with limited resources relative to other farmers)—already seen as among the most vulnerable to climatic variability and uncertainty (Morton, 2007, Oppenheimer et al., 2014)—will likely need to adapt livelihood portfolios and land use systems to ensure food security in the short term and equitable and sustainable growth and poverty reduction in the medium and longer term (Rosenzweig et al., 2014, Smith et al., 2014). This confluence of development transitions and climate change necessitates in-depth empirical studies that examine the mechanisms by which smallholders manage and adapt to multiple intersecting risks (Agrawal and Lemos, 2015, Burnham and Ma, 2016). In particular, research is needed on how agricultural intensification policies enable and/or constrain smallholders from adapting to climate change (Lal et al., 2015).

Smallholder vulnerability to climate change is shaped by interacting environmental, social, political, and economic processes operating at multiple scales (Eakin, 2005, McCubbin et al., 2015, Nyantakyi-Frimpong and Bezner-Kerr, 2015, O'Brien and Leichenko, 2000). These multidimensional stressors and risks influence adaptation decisions (Bassett and Koné, 2017, Forsyth and Evans, 2013, McDowell and Hess, 2012, Milman and Arsano, 2014). Recent work has shown how local institutions—formal and informal rules and norms—can mediate these decisions (Agrawal, 2010, Shinn, 2016, Wang et al., 2013). However, much uncertainty remains concerning smallholders’ current and future mechanisms of adaptation and how these strategies may shift in response to climate change interventions and development policies (Burnham & Ma, 2016). Conceptual and empirical work clarifying linkages between development policies and smallholder capacities to adapt to socio-environmental change is particularly limited (Eakin and Lemos, 2010, Lemos et al., 2016), as is research on how social and political economic relations shape vulnerability to climate change (Turner, 2016). As a result, climate risk management is seldom incorporated into development policies, leading to limited effectiveness in equitably addressing adaptation challenges (Agrawal & Lemos, 2015). This has also meant little opportunity to address uneven power relations and social inequalities that may be at the root of these vulnerabilities (Bassett and Fogelman, 2013, Ribot, 2011).

It is essential to investigate how development intersects with other social-environamtal processes to enable and/or constrain people from adapting to climate change (Eakin, Lemos, & Nelson, 2014), or their adaptive capacity, which is produced at the interface of political ecnomic structures and household agency (Clay, 2018a). Adaptive capacity comprises the assests that people can draw on to change as well as the power, knowledge, flexibility, and agency to decide whether to change or not (Cinner et al., 2018). Recent work suggests that local institutions are key mediators of adaptive capacity (Birkenholtz, 2012, Eakin and Lemos, 2010, Wang et al., 2013). Some have encouraged differentiating between “specific” adaptive capacities (those explicitly addressing climate-related risk) and “generic” capacities (i.e. general livelihood components and the structural constraints leading to inequalities in education, income, or political autonomy) and examining positive and negative feedback between the two (Eakin et al., 2014, Lemos et al., 2013). Others suggest an ‘adaptation pathways’ approach to visualize how the dynamic, multi-scalar processes shaping vulnerability and adaptation coalesce over time and shape adaptation trajectories (Fazey et al., 2016, Wise et al., 2014). In contrast to temporally-static framings, adaptation pathways work illustrates fluidity in decisions and helps uncover how certain adaptations come to stabilize over time (Burnham & Ma, 2017). In many ways, these assessments of social-ecological dynamics resemble livelihoods perspectives (Bebbington, 2000, King, 2010, Scoones, 1998), which are relatively unexplored frameworks for considering climate change adaptation (Clay, 2018a, Scoones, 2009).

In this paper, we build upon this work on adaptation pathways and the sub-components of adaptive capacity. We investigate how adaptive capacities are unevenly shaped amid development transitions in rural Rwanda and how this leads to differential adaptation pathways for smallholder households. This paper illustrates how development interventions that emphasize input-led agricultural intensification have uneven outcomes for smallholders' capacities to adapt to climate change and other risks. In so doing, this research speaks to the need to reformulate development policies in ways that can address deeply-rooted social inequalities and facilitate “transformative adaptation” (Bassett & Fogelman, 2013, p50; see also, Pelling, 2011). The paper presents the results of a mixed-methods case study of four communities in rural Rwanda. Two questions frame our analysis: (1) How do cross-scalar social, political economic, and environmental processes converge to shape smallholders’ adaptive capacities? and (2) How do these uneven capacities lead to differential adaptation pathways?

In addressing these questions, we contribute empirical reinforcement and conceptual clarity on how specific and generic components of adaptive capacity intersect. We find that the implementation of an input-led agricultural intensification program in Rwanda has deactivated social institutions of climate risk management (e.g. autonomy over cropping decisions and seed sharing), leading to diminished specific adaptive capacities for many households. In their place, new adaptation options have emerged for some households in the form of (a) commercial agriculture with associated input access and crop insurance, and (b) planting woodlots on surplus land for charcoal and timber. However, a consequence of development programs in Rwanda is that these adaptation strategies are only available to households with certain livelihood portfolios. To build and employ these alternate specific capacities, smallholders must negotiate new social institutions, a prerequisite for which is land, labor, and stable nonfarm income. We find that the majority of households that lack these entitlements are pulled deeper into poverty with each successive climatic shock.

## 2 Research framework and background

To investigate how adaptive capacity is differentially built amid development transitions in rural Rwanda, we integrate recent insight on the multiple components of adaptive capacity and adaptation pathways with livelihoods perspectives, which have long attended to multi-scalar processes of agrarian change within which smallholders make decisions (Scoones, 2009). We focus on adaptive decision-making in the context of Green Revolution-inspired agricultural intensification policies and related programs of rural transformation in Rwanda. Our analytical approach enables us to 1) visualize the array of social-environmental processes that interact to shape smallholders’ adaptive capacities and livelihood adaptation options and 2) identify patterns in how these adaptive capacities arise unevenly in accordance with pre-existing livelihood portfolios and social inequalities. This *adaptive livelihoods* approach (see Clay, 2018a for further description) allows us to chart how development transitions intersect with climate change to give rise to unequal adaptation pathways: trajectories of accumulation for some, maladaptive paths to further impoverishment for others.

### 2.1 Smallholder adaptation and development

Smallholders’ abilities to manage climatic risks can be shaped by how well they navigate shifts in agricultural policy (Eakin, 2005) and there is arguably a need to make climate risk management a central feature of development planning (Agrawal & Lemos, 2015). To create opportunities for equitable adaptation to climate change, it is increasingly recognized as essential to address deeply rooted structural inequities and power differentials that have implications for distributive justice (Bassett and Fogelman, 2013, Pelling, 2011, Shinn et al., 2014, Turner, 2016, Tschakert et al., 2013). To illuminate such power differentials and how they shape adaptation options, burgeoning work focuses on the lived experiences of smallholders via in-depth empirical assessments of adaptation processes (Burnham and Ma, 2017, Eakin et al., 2009, Feola et al., 2015, Nightingale, 2017, Shinn, 2016).

For one, this work has demonstrated that top-down technocratic development and adaptation interventions can mismatch with, overlook, or undermine local-level processes that are vital to adaptive capacity (Burnham and Ma, 2017, Rasmussen, 2018, Sovacool, 2018). More generally, these studies show that vulnerability and adaptation are relational: rather than being characteristics of individual actors, they are produced through relationships with other actors, relationships that are context-dependent and in flux (Turner, 2016). As a lens to consider how vulnerability and adaptation emerge at the interface of political economic structures and smallholder agency, some suggest considering the role that institutions play in adaptation processes (Birkenholtz, 2012, Carr, 2008, Rasmussen, 2018, Yeh et al., 2014). Institutions—that is, formal and informal rules and norms “that humans use when interacting within a wide variety of repetitive and structured situations” (Ostrom, 2008, p. 24)—can be crucial sites of negotiating adaptation actions (Agrawal, 2010).

A key emerging theme in this literature centers on understanding how adaptive capacity to climate change is unevenly shaped amid socio-environmental change (Clay, 2018a). In this vein, studies have demonstrated that differential asset bases can lead to divergent adaptation strategies among households in a single community (Jain, Naeem, Orlove, Modi, & DeFries, 2015) and that power asymmetries and the disintegration of social institutions can intersect with climatic events to shape adaptive capacity (Engle & Lemos, 2010). For example, research has shown that adaptive capacity can be influenced by shifting social norms governing common property resource grazing space during drought in East African rangelands (Goldman & Riosmena, 2013) and by local power dynamics that authorize subsistence use of wetlands during flood events in Botswana’s Okavango Delta (Shinn, 2017). Within institutional change, specific adaptive capacities (those explicitly mitigating climate risk) as well as generic capabilities corresponding to livelihoods, assets, education, and political power are recognized as key to building adaptive capacity to climate change (Eakin et al., 2014, Lemos et al., 2013).

### 2.2 Adaptive livelihoods: Building adaptive capacity

This recent emphasis on generic capabilities and building adaptive capacity calls to mind a rich tradition of livelihoods approaches, which have long focused on capabilities and resource governance institutions (Nussbaum and Sen, 1993, Sen, 1999, Leach et al., 1999), but which have seldom been employed to consider climate adaptation (Scoones, 2009). For instance, Nussbaum and Sen (1993, p. 3) assert that the “capability of a person refers to the various alternative combinations of functionings, any one of which (any combination, that is) the person can choose to have.” The work of Leach et al. (1999, p. 232) builds upon this by advocating for an environmental entitlements framework to explain “how the consequences of environmental change in general, and access to and control over natural resources in particular, are also socially differentiated.” Methodologically based in assessments of these capabilities, livelihoods approaches provide a useful contribution to adaptive capacity assessment by enabling appreciation of the diverse factors that shape heterogeneous opportunities for people. Put differently, capabilities—and fundamental livelihood possibilities—are shaped through power-laden, context specific processes (King, 2010).

Importantly, livelihoods are not simply a function of having enough economic capital to respond to social-environmental change; rather, what is at stake is the possibility to leverage these resources and relationships, or have the *agency to generate alternative adjustments*, as needed (Bebbington, 2000). Our analytic draws on livelihoods frameworks (Bebbington, 1999, Ellis, 2000, Scoones, 1998) in that it positions generic capacities as associated with capitals (human, natural, physical, social, and financial). In turn, the process of translating those generic capacities into specific climate risk mitigation mechanisms resembles entitlements/capabilities (i.e., abilities to access and utilize resources), which livelihoods frameworks posit are embedded in institutions (Leach et al., 1999). Drawing from livelihoods frameworks encourages us to see adaptive capacity not as a quantifiable entity but as a complex process (Clay, 2018a).

More specifically, we approach adaptive capacity as a process that unfolds in places over time by building from recent work on adaptation pathways. Doing so helps fill a crucial gap concerning how some groups and individuals come to have more or less adaptive capacity than others. Adaptation pathways research illuminates how adaptation decisions emerge and build on one another over time and how adaptation trajectories can vary by social groups and across levels of organization (e.g. household, community) (Butler et al., 2014, Fazey et al., 2016, Wise et al., 2014). By de-emphasizing assessments of adaptation ‘successes’ or ‘failures’ and instead highlighting fluidity in adaptation processes, this work depicts how seemingly discrete adaptation decisions coalesce into broader trajectories of change (Fazey et al., 2016). For example, Burnham and Ma (2017) demonstrate that smallholders in central China have gravitated towards maize cultivation because the crop permits livelihood diversification, is consistent with social norms surrounding agri-environmental management, and is perceived as resilient to environmental and market risks. Adaptation pathways can also trend towards *maladaptation* (Wise et al., 2014). For example, Carr (2008) shows that smallholder agricultural diversification strategies can persist even with sub-optimal household-level outcomes due to their coherence with pre-existing gender roles.

In this paper, we consider how adaptation pathways emerge amid development transitions by investigating the interplay among specific and generic adaptive capacities. This adaptive livelihoods approach encourages us to consider how adaptive capacity is unevenly built. That is, how individuals, households, and social groups come to differ in their abilities to adaptively respond to climatic variability and change. As we discuss below, this approach helps understand links between development and smallholder adaptation to climate change. Specifically, we assert that assessments of adaptive capacity can be more constructively framed by considering development policies and programs as imbuing complex negotiations and interactions among diverse actors that shape adaptation pathways. We demonstrate this approach through a case study of how differential adaptive capacities emerge in Rwanda amid development transitions associated with the ‘African Green Revolution’ (UN, 2004).

### 2.3 Africa’s Green Revolution in Rwanda

Over the past decade, development policies across Sub-Saharan Africa (SSA) have come to re-center on smallholder agriculture as an engine of economic growth. In hopes of catalyzing an African Green Revolution (World Bank, 2007), major development actors (e.g. the World Bank, the United States Agency for International Development, and the United Nations) and national governments have made substantial investments in agricultural intensification, (Jayne et al., 2010, Moseley et al., 2015). As in earlier Green Revolutions in Mexico and Asia, contemporary agricultural intensification programs throughout SSA center on smallholders, aiming to encourage households to adopt high-yielding varieties of maize, rice, and wheat and to increase application of agrochemicals, which these varieties often require to achieve optimal productivity. Africa’s Green Revolution has thus far taken shape as a top-down enterprise, with governments and development agencies incentivizing and sometimes enforcing the increased adoption of technologies (Gengenbach et al., 2018, Moseley et al., 2015, Clay, 2017). Not only do intensification policies aim to sustainably end hunger and malnourishment—which have abated very little in SSA compared to other regions—they illustrate a renewed development emphasis on agriculture as an engine of overall economic growth (Rashid et al., 2013, World Bank, 2007). As with earlier Green Revolutions, land and technology are identified as the limiting factors (Jarosz, 2014). Increased inputs (such as hybrid seed, chemical fertilizers, and pesticides) and agroengineering (terracing, draining wetlands, and irrigating fields) are thus positioned as essential to shore up ‘yield gaps’ and thereby curtail further expansion of agricultural land into biodiverse areas (Foley et al., 2011, Lobell et al., 2009, Tilman et al., 2002).

However, political economic and agroecological contexts in contemporary SSA poorly resemble those of 1960s and 1970s Asia. In contrast to consistent national markets and robust state-led subsidy programs that facilitated Green Revolutions in India, for example, decades of neoliberal reforms in SSA have meant weakened state involvement in agriculture and dependence on erratic global markets (Bezner Kerr, 2012, Dorward and Kydd, 2004). There is growing evidence that input-based agricultural intensification has done little to meet pro-poor development goals in SSA. Although aggregate crop yields may have increased in some places, national-level food production figures say little about local-level food access (Bezner Kerr, 2013). Technology-driven intensification policies appear to have done little to address longstanding unevenness in land and resource access among smallholders, resulting in exacerbated malnutrition and inequality among some rural communities while large-holders, urban populations, and agribusiness benefit (Bezner Kerr, 2012, Moseley et al., 2010, Moseley et al., 2015). Moreover, increased yields in rural areas do not guarantee the emergence of nonfarm employment opportunities (Haggblade, Hazell, & Reardon, 2010) and increased commercial activity is not universally beneficial across the socially diverse range of smallholder farmers (Clay, 2018b, Gengenbach et al., 2018). To speak to the viability of intensification policies for pro-poor rural development in SSA, local-level research is needed to scrutinize links between intensification, yield increases and poverty reduction (Abro, Bamlaku, & Hanjra, 2014).

With its Crop Intensification Program (CIP), Rwanda is an archetype of the New Green Revolution in SSA. In 2007, the country was the first to sign a compact with the Comprehensive Africa Agriculture Development Program (CAADP), a pan-African framework of agriculture-led growth. Since 2004, Rwanda’s Strategic Plans for the Transformation of Agriculture (PSTAs) have systematically transformed rural landscapes and livelihoods through land reform and technology adoption (Clay, 2017, GOR, 2004, MINAGRI, 2012). Striving to become a middle-income country by 2020, the GoR aims to convert largely subsistence-based smallholders to professional farmers. With its Land Policy (since 2006) and CIP (implemented since 2007 in pilot phases and countrywide since 2010), the Government of Rwanda (GoR) and development partners have formalized land tenure, drained marshlands, and constructed terraces throughout the country (Kathiresan, 2012). The CIP aims to increase the productivity of export-oriented crops by increasing access to hybrid seed and fertilizer, by augmenting agricultural extension services, and by compelling smallholders to consolidate landholdings with neighbors and cultivate crops selected by government authorities (MINAGRI, 2011). Crops under this ‘crop regionalization’ mandate (wheat, maize, rice, bean, cassava) were selected via a national-level exercise that considered agroecological zones and the crops’ export potential (Huggins, 2014). Contracts (*imihigo*) with higher administrative levels outline hectare targets on which to consolidate land and plant selected crops, assuring that national-level targets are enacted at local levels (Ingelaere, 2014). Routine audits at various levels further solidify the top down nature of this program. To ensure that they meet government quotas for land in the CIP: local administrators threaten noncompliant farmers with fines, uprooting crops, or appropriating land (Cioffo et al., 2016, Clay, 2017).

As a CIP report states, the program “encourages crop specialization to realize economies of scale and to orient the agricultural sector more towards the commercial market” (MINAGRI, 2012, p30). While increases in agricultural production and commercialization are seen to have played a role in driving Rwanda’s impressive economic growth (8 percent yearly from 2000 to 2012) and poverty reduction (EICV, 2015; World Bank, 2014), research also shows that these policies may exacerbate uneven land access (Dawson, Martin, & Sikor, 2016) and food insecurity (Clay, 2018b). Studies on the initial phases of the CIP found that smallholder autonomy has been constrained, especially for socioeconomically disadvantaged households (Ansoms, Walker, & Murison, 2014; Huggins, 2014, Pritchard, 2013). Studies also demonstrate that smallholders’ ability to succeed with CIP-prescribed land use practices is highly uneven—varying according to agroecolgical conditions and pre-existing resource access inequalities (Cioffo et al., 2016, Clay, 2017). The present research builds on this work by investigating how Rwanda’s Green Revolution-inspired agricultural policies shape smallholders’ capacities to adaptively respond to perceived social-environmental changes. This research is timely as the GoR and development partners are currently scaling up these policies (MINAGRI, 2012, World Bank, 2014) and given that similar policies are being widely implemented across sub-Saharan Africa (Schurman, 2018).

## 3 Methods and study sites

Empirical materials informing this study were collected via quantitative and qualitative methods carried out in Rwanda from September 2014 to December 2016. An in-depth case study was used to consider the interplay among smallholder livelihoods, land use practices, and development transitions in the context of climatic variability, uncertainty, and change. Surveys and interviews focused on smallholder resource access and decision-making within changing institutional contexts and broader social-environmental changes. Iterative phases of data collection enabled the research team to build and test hypotheses and to periodically solicit feedback from study participants. As demonstrated by other studies, empirical attention to lived experiences with climatic shocks and adaptation to perceived climate change can yield valuable insight on response pathways in the face of current and future global climate change (Engle and Lemos, 2010, Turner, 2016).

### 3.1 Study site

This study is sited in Kibirizi sector, southwest Rwanda, a mountainous region of the country, which has continually suffered from poverty and waning soil fertility, and which the National Adaptation Plan of Action for climate change identifies as high risk for both recurring heavy rain and drought (EICV, 2015, GoR, 2006). The study period coincided with numerous climatic shocks. Torrential rain and flooding occurred in 2014 and 2015; extended dry spells in 2013, 2014, and 2016 decimated harvests throughout the country (FEWS NET, 2013, FEWS NET, 2014, FEWS NET, 2016) and combined to produce what some have called “the longest drought in six decades” (Times, 2016). The study site comprises 4 *umudugudu* (smallest administrative unit, roughly translating as ‘rural settlement’) that were selected as 1) representative of rural settlements throughout Rwanda; and 2) eliciting variations in the degree and type of agricultural intensification (see Table 1). Agriculture (either own farm or working others’ land) is the principal livelihood for nearly all households, who cultivate rain-fed, mixed crop-livestock systems for both subsistence and commercial ends. Cultivation on hillsides is circumscribed by two growing seasons per year (one from September to December, the other from March to May) and restricted to marsh and valley areas during dry seasons. When possible, households diversify livelihoods to include non-farm employment such as small businesses, skilled labor in masonry or house framing, and other labor such as collecting sand or making charcoal. The CIP has been implemented in Kibirizi since 2010 and the program has been widely adopted, with over 90 percent of survey respondents participating in 2015.

**Table 1:** Four study *umudugudu* characterized in terms of type and degree of Crop Intensification Program (CIP) attributes.

*Umudugudu*	Type of Intensification	Degree of Intensification	Number of surveys	Number of interviews
A	Terraced: high Marshland: none; Other CIP: some	Highest	109	27
B	Terraced: none Marshland: some Other CIP: high	Medium-high	119	11
C	Terraced: some Marshland: some Other CIP: low	Medium-low	67	9
D	Terraced: none Marshland: low Other CIP: low	Lowest	117	24

As elsewhere in Rwanda, three variations of CIP-prescribed agriculture have been implemented in Kibirizi: terraced-hillside agriculture, unterraced hillside-agriculture, and marshland agriculture. The rules are most strict in terraced areas. A regional agronomist selects crops each season according to government targets. Although households retain land titles, farmers are strongly compelled to cultivate selected crops by threat of fine, jailing, or land expropriation if a crop is not cultivated during a growing season. On unterraced land, restrictions are somewhat eased. However, households are still incentivized to grow selected crops and discouraged from growing four crops known for their subsistence importance: sweet potato, cassava, sorghum, and a regionally important grain known locally as *ciraza*. Prior to each growing season, households submit contracts to local leaders indicating that they will grow selected crops. If they do not meet these contracts, individuals can be publicly disgraced during weekly meetings of village-level farmer associations. Cooperatives coordinate intensification activities in marshlands, with a regional agronomist making cropping decisions. While access to marshlands prior to 2005 was subject to *de facto* land rights and planted in mixed crops and grazing, access now requires paying cooperative membership (5000 RWF, or 8 USD) and seasonal use fees (2000 RWF, 3 USD) per 100 m square parcel. Seed and fertilizer for government-selected crops are distributed through cooperatives, state extension agents, and (for maize and bean) by an American company called the One Acre Fund (known locally as *Tubura*) which has partnered with the GoR. In addition, eucalyptus tree seedlings (*E. globulus* and *E. maidenii* species) are sold at subsidized rates throughout the study area in line with government aims of increasing tree cover across Rwanda.

### 3.2 Data collection and analysis

Focus groups (n = 4, one in each umudugudu; 12–20 people each; participants recruited by local leaders) and key informant interviews served to introduce the research team and develop understanding of central issues. Interviews were conducted in Kinyarwanda or French with the help of a research assistant, employing open-ended questions to query changes in livelihoods, policies, and rainfall patterns over the past twenty years. Two primary concerns emerged surrounding agricultural livelihoods: climatic shocks have increased in magnitude and frequency and the CIP has altered land use decision-making by reducing household autonomy over crop selection and access to inputs. These issues informed subsequent research phases.

Second, the first author conducted a structured household survey from November 2014 to March 2015 with an adult male or female representative of all available households (households deemed unavailable if not present on three visits on separate days^1^) in the four *umudugudu* (428 households out of approximately 500 total) by a team of four Rwandan research assistants. In addition to documenting household demographics, assets, livelihoods, and food security, questions focused on land use, the influence of Rwanda’s CIP and broader agricultural policies on decision-making and crop yields, and changes in resource access, institutions, and climate. For questions addressing political economic and agricultural changes, a 10-year timeframe was used; this is supported in literature on agrarian change in SSA (Guyer, 1997). All surveys were conducted within respondents’ home compounds and administered in Kinyarwanda (1 h 56 min average per respondent). Third, a structured parcel-level survey (administered July 2015 to October 2015) was completed with all households that had operational land holdings (402 households and their 3017 parcels), meaning that 26 landless households were excluded. Questionnaire development and administration followed the same protocol as above with the addition of four local research assistants (secondary school graduates) who were trained in calculating field area and use of GPS units. All parcels less than 30 min’ walk from the homestead were visited, otherwise location was indicated on a map. The questionnaire included 30 questions at the household level (number and location of parcels, general challenges to agriculture, seed and labor availability, etc.) and 70 questions for each parcel operated, including general parcel characteristics (fertility, soil type, tenure status, years operated, etc.) and crop choice, yields, and challenges affecting yield for the previous three seasons.

Fourth, in-depth semi-structured interviews were conducted with 36 male and 36 female respondents (April 2015 to August 2015) from randomly-selected households. Each of these 72 interviews involved three members of the research team: one asked questions in English, another translated between English and Kinyarwanda, and a third took detailed notes. Meetings immediately following each interview ensured that details and overall meaning were accurately recorded. Interviews averaged 1 h 20 min and concentrated on changes in climate, land use, livelihoods, household agricultural labor, agricultural challenges, and perceptions and impacts of government policies. Fifth, unstructured interviews were conducted (October 2014 to December 2016) with 32 individuals representing key institutions involved in agro-ecosystem management, including leaders in six umudugudu, five mayors at ‘cell’ and ‘sector’ levels, agronomists, state employees overseeing land tenure issues, cooperative presidents, input distribution companies, health workers, local development officers, and security officials, among others. Questions pertained to contemporary and historical political, economic, environmental, and agricultural aspects of the study area and broader region, including the implementation of Rwanda's CIP and perceptions of climate change.

In-depth interviews complement quantitative data with rich detail on power relations and the effects of institutional change. As a case study, this work does not consider regional differences. However, it provides a comprehensive picture on how development transitions intersect with broader social-ecological change, enabling visualization of the complex pathways by which smallholders weave together livelihood and land use strategies within social and biophysical contexts that vary markedly within the study site. Data from household and parcel surveys were analyzed using SPSS. Tests included descriptive statistics, bi-variate comparison of means, bivariate correlation, Chi-Square, multivariate analysis of variance (ANOVA), and other multivariate regressions. Interviews were coded and analyzed qualitatively using ATLAS.ti software. An initial round of coding was done and refined into a codebook that was employed in a second round of coding, enabling sub-themes to emerge. The coding strategy reflected attempts to account for relationships deemed statistically robust as well as to further explain relationships that remained unclear following statistical analyses.

## 4 Results

Our results on the processes by which adaptive capacity is unevenly built are organized as follows. We first depict household differences in terms of livelihood capitals. We then explore the multidimensional processes of adaptation, demonstrating how livelihood capitals and capabilities – and entrenched social inequalities – are woven into adaptation pathways. We show that smallholders must draw on their generic capacities to implement risk management mechanisms, leveraging multiple capitals to maintain production of subsistence crops while also adopting two new land use strategies: 1) commercial agriculture in line with the state’s vision of modern farming systems, including valley and marshland cultivation during dry seasons; and 2) woodlots for timber and charcoal production. In discussing the mechanisms of these adaptations, we illustrate how smallholders come to differ in their capacities to adapt amid social and environmental change, the role of social institutions in this process, and how differential capacities lead to uneven livelihood adaptation pathways.

### 4.1 Capitals and climate change

Quantitative analysis of survey data reveals vast differences in livelihood capitals (shown in Table 2) among households within the study sites. Results from a two-step cluster analysis (hierarchical and non-hierarchical using continuous and categorical variables) are displayed in Table 2 along the three pillars that informed the cluster analysis: agricultural assets, income streams (a proxy for nonfarm livelihood diversification), and homestead characteristics (viewed as a key indicator of wealth in Kibirizi). These livelihood groups are used in subsequent analyses alongside other variables to consider the effects of institutional change on adaptive capacity and the ways in which livelihood capitals are employed to adopt new land use and livelihood strategies amid variable and changing climates.

**Table 2:** Household livelihood capitals organized into four groups through cluster analysis. Means and percentages displayed by group for the 8 variables that comprised the cluster analysis. Analysis of variance (ANOVA) finds significant across-group variation for all 8 variables at p < .05 level.

		Agriculture			Income streams		Homestead		
Generic capacity level	N	Land	Cows	Pigs, goats or sheep	Non-farm income (RWF)	Farm-labor income (%)	Concrete floor (%)	Brick or cement walls	Electricity
Low	117	0.12	0.1	0.5	5252	91.9	0.9	0.9	1.7
Medium low	137	0.29	0.3	0.8	21,268	69.4	2.7	3.0	3.0
Medium high	128	0.65	0.6	1.2	147,576	52.9	8.6	15.6	13.6
High	49	1.54	1.3	2.1	611,676	34.4	24.5	36.6	26.7
All groups	431	0.51	0.5	1.0	120,640	71.8	6.3	0.5	1.0
Sig.	–	0.000	0.000	0.000	0.000	0.000	0.000	0.000	0.000

### 4.2 Adoption of commercial agriculture

#### Context of changing climate

As discussed above, the years surrounding the period of this study were marked by erratic rainfall, including several seasons where rains ended early or began late, seasons where rain stopped for long stretches during key stages of crop development, and seasons with torrential rain and flooding that destroyed crops. Focus group and institutional interviews early in this study revealed that adverse climate and soil fertility are major impediments to agriculture. Precipitation patterns are widely viewed as less predictable than they were 10 or more years ago. Study participants commonly spoke of rains that used to occur but no longer do, or changes to their start and end dates. For example:

‘It can’t happen again anymore… many years ago rains fell at times that allowed harvest and then land preparation for the next season…. now it keeps changing, it can begin in September or October, we just don’t know.’ (focus group, October 2014)

‘Nowadays it is starting to rain in April but in past years it was raining in February, where farmers used to plant wheat in March. The rain used to stop in June but now it stops in May.’ (Interview, May 2015)

‘Everything has changed with the rain and hunger has been the result. Before…production was sufficient. Then there was more rain. Now sometimes seeds do not even come up.’ (focus group, October 2014)

In addition to changing climate, there is widespread acknowledgement of two major changes to land management throughout Kibirizi. First, four crops that were once extensively cultivated (sweet potato, cassava, ciraza, and sorghum) are now effectively forbidden by the GoR. According to respondents and local leaders, the chief reason for this shift is that these crops are not part of the CIP due to their low market values. This is in spite of wide agreement that these are among the most drought and flood tolerant crops, especially when compared to much riskier CIP crops. We also explored with producers the issues of adopting the CIP in terms of institutions of climate risk management. Intersections between the government’s intensification program and issues of climatic uncertainty in Kibirizi were evident early on and throughout this study. When asked about any difficulties of adopting CIP land use practices, study participants often couched responses in terms of climate. For example:

‘The program of land use consolidation is here but because of drought seeds did not grow. We put seeds in the soil but they did not grow. We recognize that land use consolidation is important but due to climate change we are losing. There is no means for irrigating during drought.’ (focus group, October 2014)

#### Changing risk management institutions

Despite increasingly precarious climatic conditions, Kibirizi smallholders have been pressed to rapidly adopt an external vision of intensive agricultural practices in line with the CIP. Adoption is widespread, with over 93 percent of survey respondents taking up CIP land use practices in 2015. To better understand the uneven outcomes of this institutional change, we situate Rwanda’s program of agricultural commercialization in a shifting social, environmental, and political economic context within which smallholders manage risks and make adaptive decisions. While the aim of the CIP is to intensify and commercialize agriculture to create more resilient smallholder production systems, we find that the program has had corollary shifts in local social institutions of smallholder climate risk management that, for many, actually decrease resilience to droughts and heavy rain. As observed elsewhere in SSA, smallholders in Rwanda employ various climate risk management strategies that include *ex-ante* (decisions made prior to a growing season to minimize risks), *in-season* (responses to perceived and forecast weather conditions that drive adjustments to crops or cultivation practices), and *ex-post* (buffering negative impacts of climate shocks that already occurred) (see Cooper et al., 2008, Waha et al., 2013 for this typology). Surveys and interviews revealed that smallholders in Kibirizi have traditionally employed a range of strategies (summarized in Table 3). These include four principal *ex-ante* land use strategies to buffer risks of uncertain climate: a) intercropping several crop varieties within a parcel; b) cultivating parcels at multiple locations (including upper, mid, and lower hillside as well as valley and marshland); c) preparing fields and acquiring inputs ahead of time such that seeds can be sown after first rains; d) selecting low-risk crops. Respondents noted two principal *in season* risk management strategies: a) adjusting crops in response to perceived and forecast weather conditions such as planting short-maturation crops like sweet potato rather than maize if rains are delayed; and b) avoiding investing further inputs in fields that are seen as likely to fail following adverse climate conditions (for example, not weeding or applying fertilizer after inconsistent rains in the first months of a growing season). Some strategies extend across seasons; for example, social networks of seed sharing permitting cultivation in a season after drought even if household seed stores are depleted.

**Table 3:** Specific capacities for adapting to and coping with climate shocks in Kibirizi, their related social institutions. Followed by new specific adaptive capacities and new institutions regulating them.

Type of specific adaptive capacity	Example of specific adaptive capacity in Kibirizi	Related social institutions	New specific adaptive capacities	New institutions
*Ex-ante*	Intercrop fields	Household decision-making autonomy	Commercial agriculture generates money	Govt crop decisions
	Many field locations			Cooperative/Tubura
	Select low risk crops		Convert crops to woodlots	Sale of low fertility land
	Prepare fields early	Available labor	Pay for field preparation	Low-cost labor supply
	Plant marshland in dry season	Common property	Access valley/marshland	Cooperatives/cash

*In season*	Adjust crop choice	Household decision-making autonomy	Crop insurance	Cooperative/Tubura
	Abandon field		Cooperative gives seed to replant	Cooperative/Tubura
	Seed sharing (for next season)	Social capital	Seeds to replant from cooperatives	Cooperative/Tubura

Ability to employ this range of strategies has traditionally comprised Kibirizi smallholders’ specific adaptive capacities. These capacities are facilitated by various social institutions operating at multiple levels. Many of these institutions are currently in flux due to intersecting social-environmental changes (summarized in Table 3). Several key social institutions pertain to flexible decision-making at household and parcel levels concerning which crops to grow. Respondents noted that norms of land-use flexibility are deeply engrained in Kibirizi. However, the state’s crop regionalization and land use consolidation programs have sharply eroded household autonomy over cropping decisions: 75 percent of survey respondents indicated that the government primarily makes cropping decisions. In short, this means that smallholders are increasingly compelled to grow crops selected by local leaders for their supposed economic value rather than selecting crops themselves according to household needs, agroecological features of their parcels, and climate risk. As interview respondents noted: ‘Decisions have changed because of the government program and performance contracts;’ ‘The plots are under land use consolidation where one selected crop by local leaders is grown;’ ‘All decisions are coming from our local leaders.’

One result of these governance changes is that 91 percent of households now cultivate fewer crop varieties than they did ten years ago. Not only does this eradication of flexible decision-making institutions debilitate *ex-ante* capacities, it also has implications for *in-season* capacities. As discussed above, smallholders must submit contracts (which both bind producers to the state’s selected crops and allow calculation of the amount of seed to be delivered to particular rural areas) months ahead of growing seasons. This affords little leeway to adjust cropping decisions in response to climate cues. Constraints to *in-season* capacities are most pronounced in terraced areas, where producers risk land appropriation if they choose not to cultivate their holdings for a season. Negative effects associated with these policy changes are widespread, as summarized in the following responses: ‘We don't have good production and we lose all the crops planted;’ ‘we harvest less and we are obligated to buy additional food on market;’ ‘this program is the main cause of hunger because we are growing just one crop.’

Another risk management strategy that 64.4 percent of respondents noted is preparing fields ahead of time so that when rain does arrive seeds can be planted without delay. However, due to challenges in accessing seeds and labor on time, 35.3 percent of respondents report planting late during the preceding year. Difficulties planting fields on time appear to be increasingly common for several intersecting reasons associated with development transitions in Rwanda. For one, hybrid seed (maize, wheat, and bean) for CIP crops, along with inputs that are required for these crops, must be purchased. Privatization of seed and fertilizer distribution has decreased ability to access seed, since seed must be purchased at steep interest rates, a risk that many smallholders are unwilling to accept. Further, commercialization of input networks appears to have led to a decline in local seed sharing practices. These seed access challenges converge especially among those lacking nonfarm income, who must first work in others’ fields before they prepare their own fields in order to afford seed and other inputs. Moreover, respondents commonly noted that government seed deliveries are frequently delayed, and that they are not permitted to plant other seed but must instead delay planting fields until seed arrives and therefore risk missing a crucial period in the growing season.

Government-implemented institutions associated with carrying out the CIP have in some ways replaced more locally-based institutions. These could in theory correct for the diminished specific adaptive capacity caused by the suppression of traditional risk management institutions, perhaps thereby easing the transition to intensive production of commercial crops. For example, distribution of seed and fertilizer for CIP crops has been privatized and is now being carried out by agricultural cooperatives and by Tubura. Tubura provides maize seed and chemical fertilizer on credit, charging 19 percent interest. While the company provided a rebate of 50 percent on these loans following a nationwide drought, many respondents noted that for local or regional climate shocks they offer no such assistance. Similarly, cooperatives do not provide a rebate following localized flooding that frequently destroys crops. As such, these institutions are seen by many respondents as limited in their risk mitigation effectiveness. Moreover, they are not even accessible to households without a steady cash flow, who see Tubura’s high interest rates as potentially exacerbating risks in the event of climate shocks or pest outbreaks. These reservations are reflected in uneven Tubura membership, shown in Table 4. While nearly 60 percent of the wealthier households are members, only 21 percent of the poorest households are members of this institution.

**Table 4:** Results of ANOVA and Chi-Square, displaying variation in membership rates in key institutions associated with the CIP as well as the percent of CIP land where yield-reducing challenges were indicated.

	Tubura Member		Cooperative Member		Land in CIP		
Generic capacity level	N	Percent of group	N	Percent of Group	Percent total land	Percent of parcels	Percent CIP land with challenges
Low	21	20.8	22	19.0	34.4	34.0	36.1%
Medium low	52	40.3	32	23.7	30.3	32.2	29.8%
Medium high	61	50.0	25	19.8	24.8	29.4	21.6%
High	28	58.3	14	28.6	19.0	30.6	14.7%
All	162	40.5	93	21.8	28.3	31.6	26.3%
Sig.	0.000	0.104		0.366	0.000		

Given that they are accessible only to households that already have substantial generic capacity, reliance on institutions such as Tubura does not confer inclusive risk mitigation. Moreover, lack of access to the core institutions of the CIP among the poorest households creates a paradox in that these households are relatively more invested in the CIP than are their wealthier neighbors by proportion of land area. As shown in Table 4, the poorest households have more than one third of their land under CIP management, while the wealthier households have less than one fifth of their land set aside for the program. In other words, those with less institutional support to make the transition to commercial agriculture are, often unwillingly, under greater pressure to alter production practices in line with the government’s vision. Many interview respondents among lower capacity households expressed challenges in how institutional change and climatic shocks have intersected over the past five years. For example, an elderly male smallholder explained:

“They told us not to grow sweet potato and cassava and only to grow wheat and beans, and farmers were dying of hunger. It cost between 5,000 and 6,000 Frw for a basket of sweet potatoes [about ten times the typical price].”

**Interviewer:** “Is it the government policy that caused sweet potatoes to become so expensive?” ‘No. it is because of two reasons. The first reason is that there was too much sun [drought] and people could not cultivate sweet potatoes. Then after rain those who did plant sweet potato had them uprooted by local leaders.’

#### Acquiring marsh and valley land

Marshland in Kibirizi has become less accessible with the transition from ownership by local associations to more formal cooperatives. Many respondents noted that they were once part of relatively informal associations that regulated marshland use prior to 2005. At that time, people were cultivating as they liked in marshlands, often intercropping sweet potato with maize or other crops, a strategy seen to increase resilience of the plots to flooding. Now, marshlands are part of the CIP and only one crop is grown at a time. Respondents expressed frustration about this transition, noting the challenge of joining cooperatives due to high cost and the fact that cooperatives quickly reached a cap after which further members were not admitted. They lamented the loss of decision-making power over crop choice and spoke of frequent flooding that has occurred in marshlands ever since they were drained and cultivated on a large scale in just one crop.

With marshland circumscribed by CIP cropping systems, valley land has taken on renewed importance as a dry season cultivation option. Qualitative analysis of interviews clarifies that there are several intersecting reasons for this. For one, valley land can be highly fertile and permit cultivation through long dry periods. Secondly, valleys are now the only place where prohibited but important food security crops like sweet potato and sorghum are, unofficially, allowed to be cultivated. However, this higher demand has led to escalating costs for purchasing or renting valley land. As Table 5 shows, access to valley land is uneven. As a proportion of total cropland (including woodlots), valley land comprises only around 10 percent among lower capacity households, compared to two and three times that among the two higher capacity groups. Table 5 also shows that there is unevenness in the number of different parcel locations operated and that diverse parcel locations have increasingly concentrated in the hands of higher capacity households. In short, while there is a net loss in parcel locations among lower capacity groups, the highest capacity group has seen a net gain in parcel locations.

**Table 5:** Results of ANOVA and Chi-Square, displaying differential access to valley land, parcels outside of formal CIP jurisdiction, and unevenness and change in number of parcel locations operated according to socioeconomic groups.

	Land in Valleys		Parcel Locations (1–5 locations)			Has field (s) beyond CIP	
Generic capacity level	Proportion of total cropland	Cultivate in dry season (% of group)	Number of locations	Locations decreased	Locations increased	N	Percent of Group
Low	11.7%	29.2	1.8	19.1	13.6	47	46.1
Medium low	8.7%	37.6	2.0	16.4	17.2	61	48.8
Medium high	18.0%	43.2	2.2	12.2	17.9	68	58.6
High	28.1%	57.1	2.5	8.2	22.4	30	68.2
All	14.6%	39.3		14.9	17.1	206	53.2
Sig.	0.001	0.006					0.039

This discrepancy is also reflected in the proportions of each group operating agriculture during the dry season and those with access to fields beyond the formal reach of the CIP (Table 5). Table 6 further clarifies other factors associated with access to these key land types. Intensity of CIP activity is negatively related to accessibility of places to plant CIP crops, suggesting that where the program is most strict, access to places to plant food security crops is also lowest. Relatedly, CIP intensity, in combination with lower household capacity and numbers of field locations stands out in the number of climatic and other challenges experienced by households on CIP land. Similarly, households living in areas of higher CIP intensity are more likely to run out of food earlier than they were ten years ago (‘consume household produced food earlier’ in Table 6). The implication of this finding in terms of specific adaptive capacity is that even while valley land is becoming more important as a risk management strategy, it is becoming further out of reach for households living closest to the margin.

**Table 6:** Results of binary logistic regression and of multivariate regression analysis of land use access and outcomes associated with the adoption of CIP agriculture in Kibirizi.

Factor or coefficient	Place to plant food security crops		Household operates season C		Consume household produced food earlier		Percentage of challenges on CIP land	
	Sig.	B	Sig.	B	Sig.	B	Sig.	B
CIP Intensity	0.000^***^	−0.503	0.988	−0.002	0.003^***^	0.33	0.098^*^	0.015
Generic capacity level	0.068^*^	0.372	0.014^**^	0.402	0.219	0.231	0.000^***^	−0.061
Gender of HH head	0.396	−0.284	0.443	−0.23	0.199	0.447	0.677	−0.011
Age of HH Head	0.722	0.003	0.113	−0.012	0.851	−0.002	0.126	−0.001
Percent OffFarm work	0.042^**^	1.217	0.578	−0.269	0.011^**^	1.431	0.159	−0.064
Percent Nonfarm work	0.596	−0.488	0.306	−0.856	0.105	−1.457	0.441	0.064
Number of parcels	0.436	0.06	0.934	−0.005	0.175	−0.089	0.783	−0.001
Field locations total	0.609	−7%	0.645	−6%	0.758	−4%	0.000^***^	−0.046
Livestock	0.111	−0.464	0.492	0.16	0.002^***^	−0.798	0.437	0.017
Constant	0.052	1.619	0.584	−0.387	0.635	−0.383	0.000	0.548

### 4.3 Increased adoption of woodlots for charcoal

Woodlots are widely seen to have proliferated in Kibirizi over the past ten years. Over 74 percent of survey respondents noted that trees have increased in their umudugudu during this period. This is due to the perceived resilience of woodlots to climatic shocks and their importance as a source of cash once the wood is harvested and sold for charcoal or for construction. In the minds of many study participants, the rapid adoption of woodlots is closely linked to *Ruchamakara* (meaning “charcoal making hunger”), one of the worst famines in the past century. The Ruchamakara hunger period is understood to have arisen following three growing seasons of markedly low yield due to adverse climatic conditions. Though the order of climate shock events is not completely clear, most respondents indicated that it began with a prolonged period of drought that negatively affected two seasons. This drought is said to have been followed by unusually heavy rains in a third growing season. The prolonged duration of this hunger period reportedly caused some households to migrate, some individuals to perish, and a great many to participate in the production of charcoal. Charcoal making, as the name of the hunger period would indicate, was widely seen as the only recourse for households to purchase food from market in the wake of three failed harvests. Ruchamakara occurred around the year 2000, and stories still circulated of even stumps being cut out of the ground and processed in charcoal kilns. Respondents in Kibirizi also clarified that there are structural factors underlying increased tree farming. The GoR has incentivized woodlot adoption, ensuring that tree seedlings of two eucalypt species are made widely available at subsidized rates (seedlings cost 20 Frw each). Additionally, the GoR requires that all fields beyond 45 degrees (deemed too steep to farm) must be planted with trees to reduce erosion. The 45-degree slope rule is enforced by local leaders, who are often called upon to settle disputes regarding woodlots. This explicit erosion-reduction measure nests within a broader afforestation policy that has been implemented throughout Rwanda.

Converting agriculture to woodlot is an adaptive land use strategy that has thus come about through the intersection of a changing climate, declining soil fertility, increased connectivity to urban areas where charcoal is delivered, and government policy to increase tree cover. It has become a generic adaptive capacity strategy that also enhances specific adaptive capacity to climate change. Whether or not they currently owned a woodlot, respondents frequently acknowledged that they could plant trees in a woodlot, put very little work into their upkeep, and after ten or 15 years hire somebody to cut the trees into poles for home construction (if the trunks are straight enough) or charcoal (if the trunks are less straight). Both of these outputs provide substantial cash income to the woodlot owner. Perhaps more importantly, woodlots can be effectively cashed in at any time, whenever the household is most in need of money. Indeed, numerous interview respondents indicated that trees are an effective income diversification strategy, even preferable to having money in a bank.

However, adoption of woodlots is a highly uneven adaptation. Interviews clarified that woodlots are by all accounts recognized as a land use option largely available to wealthier households. And, as shown in Table 7, the percentage of total household land in woodlots correlates strongly with livelihood group. Only 26.7 percent of the poorest households own any woodlot, which is generally small and used to gather firewood, compared to 93.8 percent of among the wealthiest households, many of whom own large woodlots which are harvested for making charcoal or selling timber. Further illustrating this discrepancy, the wealthiest group of households has roughly half of their total land in woodlots while the poorest households have less than ten percent of their landholdings in woodlots. This finding is strongly supported by in-depth interviews, wherein respondents commonly acknowledged that although woodlots are the preferred climate-resilient land use, they are unable to convert to woodlots given the need to continuously produce food for their families. Indeed, while half of wealthier households report expanding woodlots in the past ten years, only 8 percent of the poorest households have planted more trees in that time period. For wealthier households, conversion to woodlots has become an important livelihood diversification strategy, as the following quotes illustrate:

‘The number of trees has increased so much. People who have land for tree planting do so.’

‘They started to grow more trees after realizing timber are being exported and trees are fetching much money. Before, people were planting only in high risk zones but now they are planting in land favorable for crops.’

**Table 7:** Results of Chi-Square and ANOVA indicating variation across household livelihood groups in the amount and proportion of woodlots among their landholdings, and whether they have expanded woodlots in the past ten years.

	Household Land in Woodlots			
Generic capacity level	Mean woodlot (ha)	Proportion of total land	Expanded woodlots in past ten years (% of group)	Trees have increased in umudugudu
Low	0.001	7.6	8.2	77.3
Medium low	0.043	11.8	16.8	75.6
Medium high	0.225	29.2	26.0	70.5
High	0.911	46.6	51.0	75.0
All		20.5	21.3	74.5
Sig.	0.000	0.000	0.000	0.668

As we explore further in the discussion, these inequalities in adoption of resilient land use strategies are symptoms of the ways in which specific and generic capacities have intersected over time to reinforce or impede adaptation options. In the next section we employ the adaptive livelihoods framework to evaluate what these findings mean in terms of the differential livelihood pathways that emerge out of uneven adaptive capacities.

## 5 Discussion: Adaptive livelihoods?

The above results demonstrate that agricultural intensification and afforestation policies in SSA intersect with changing and variable climates to shape pathways for adapting land use strategies and livelihoods. In rain-fed smallholder farming systems, the success of these policies can be predicated on how they facilitate adaptive capacities to adjust practices to mitigate increasing climatic variability and uncertainty. Our findings from southwest Rwanda suggest that land use transitions aligned with the ‘New African Green Revolution,’ a mandate of intensified, commercial-oriented agriculture, have uneven effects on the diverse range of smallholder households in the midst of global climate change. CIP-prescribed land use practices have been differentially adopted in Kibirizi, with households closest to the margin putting proportionately more of their land into the program than households with more diversified land use systems and livelihood portfolios. This has meant that smallholders most reliant on subsistence agriculture have felt the largest impact of the destabilization of local social institutions that enabled flexible decision-making in the past. The poorest households experience far more yield-reducing challenges (both climatic and non-climatic) in fields planted under CIP-specifications, challenges that are exacerbated by the degree of intensity of CIP activity, with the most strictly regulated terraced areas showing the largest negative effects on household abilities to produce enough food and to continue planting core food security crops together with their forced adoption of commercial agriculture.

By combining the adaptation pathways approach with research on the specific and generic components of adaptive capacity, this study contributes insight into how negative effects of development policies can arise through uneven access to new institutions (e.g. cooperatives and buying inputs on credit from companies) which can become necessary for managing climate risks in the absence of traditional institutions. Diversifying livelihoods to include nonfarm income has become a prerequisite for access to risk management institutions, leaving many households behind and curtailing their chances for succeeding with the CIP. In the context of these abrupt institutional changes—which compel households to adopt riskier land use practices—substantial farm assets (land and livestock) as well as sufficient capital or steady income are increasingly needed to buffer climate shocks and facilitate adaptation. While flexible land-use strategies have become less of an option for all, households with substantial landholdings (especially valley land that falls outside the CIP jurisdiction) are able to practice traditional polyculture systems of agriculture alongside intensified production. Overall, this is a diversified system that facilitates continued buffering of environmental and economic shocks and enables the continued production of food security crops. Another observable trend is that valley land, originally an important risk management resource, appears to be transitioning into a wealth accumulation platform for wealthier households, those who hold a significantly larger proportion of their cropland in valleys than do poorer households and are twice as likely to cultivate during dry seasons. In these ways, smallholders that additionally have steady nonfarm income and who operate across a variety of land types are far better positioned to absorb the risks of involvement in commercial agriculture. These households, which have higher generic capacity to begin with, can further build that foundation and thus expand their adaptive capacity to future social and environmental change. Such households can be seen to have adaptive livelihoods in that they draw on livelihood capitals in ways that enhance their abilities to manage risk and uncertainty. Conceptually, this also underscores the value of nuancing an adaptation pathways approach by considering how generic and specific capacities intersect.

By contrast, a majority of smallholder households could be classified as risk averse in that their circumstances preclude diversifying into riskier livelihoods and/or land uses. For such groups, Eakin et al. (2014) argue that development policies should aim to “build generic capacities without undermining the existing endogenous capacities for risk management: i.e., their specific capacities” (p. 3). Yet, our findings indicate that development resources are being expended in ways that provoke further inequality in household abilities to access and adopt climate risk mitigation mechanisms. The case of uneven adoption of woodlots as a climate-resilient land use strategy further illustrates how uneven adaptation pathways are produced through the interface of development policy and climate change. Faced with more uncertain climate and decreased soil fertility, households with surplus land have been able to capitalize on subsidized access to tree seedlings by dramatically expanding woodlots across their landholdings. Yet those without these bundles of resources and livelihoods (especially nonfarm work) have not adopted woodlots as a land use strategy. This uneven ability to invest in woodlots curtails adaptation pathways for many households, who instead are forced to adopt CIP agriculture on much of their land. Relatedly, the proliferation of woodlots also means less available land for rent for poorer households to produce food, which in turn is a factor in escalating land prices.

**Fig. 1:**
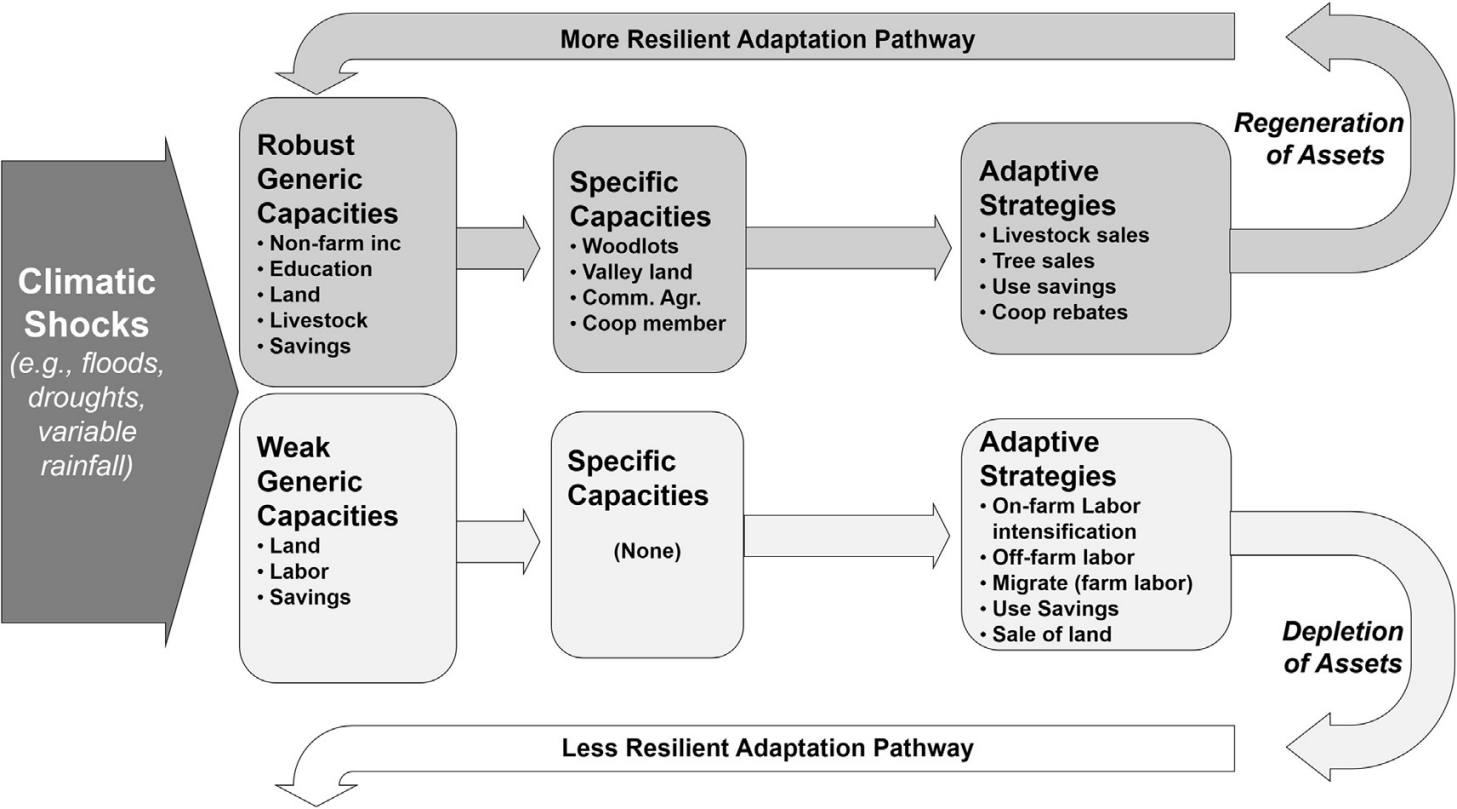
Differential livelihood pathways emerge out of uneven adaptive capacities. Institutional change prohibits traditional risk management and the resulting scenarios for wealthier households (adaptation as adoption of new specific adaptive capacities through use of livelihood capitals) and poorer households (continued poverty due to lack of options for diversifying livelihoods and land uses while also being forced to adopt commercial agriculture).

In summary, an approach that combines longer term concerns of adaptation pathways with the multiple components of adaptive capacity can help us understand how complex development processes influence households’ adaptive capacities to climate change. On top of having limited specific capacities due to these new institutional arrangements, poorer households are experiencing food shortages that stem from the challenges of implementing land use according to CIP specifications. Furthermore, without access to woodlots or enough valley land, poorer households have difficulty engaging in land use and livelihood practices that help to strengthen their fundamental livelihood capitals in the face of an uncertain climate. This demonstrates that when agricultural intensification policies focus exclusively on building general livelihood capacities through commercialized agriculture they can undermine specific adaptive capacities, leading to unwitting adoption of riskier farming practices. Opportunities can emerge for alternative risk management mechanisms. However, our findings illustrate that the adoption of these alternate specific capacities can be highly uneven as it is often rooted in fundamental livelihood capitals and capabilities (or ‘generic capacities’). As the above narrative illustrates, a series of climatically adverse seasons—as occurred from 2013 to 2016 in Kibirizi—can set in place a downward spiral that will make it even more challenging for the poorest smallholder households to climb out of poverty. These divergent synergies (negative for poorer households, positive for wealthier households) are illustrated in Figure 1.

## 6 Conclusion

This study considers household impacts and responses to climatic shocks and change (including late rain onset, drought, and heavy rain) amid broader social-environmental changes in Rwanda to understand how smallholder producers come to differ in their abilities to mitigate and adapt to climatic uncertainty, variability, and change. Findings show that certain combinations of livelihood and land use practices enable households to more effectively cope with discrete climatic shocks and adaptively respond to perceived climate changes so as to mitigate current and future risks of uncertain and variable rainfall. We demonstrate that adaptation options emerging within the context of social-environmental change are attenuated by differential adaptation pathways that embody pre-existing resource access regimes and power asymmetries. Individuals and households must navigate new institutions in order to mitigate risks of increasingly uncertain precipitation patterns and episodes of drought and heavy rain. Mitigating risk now requires either accessing institutions associated with the CIP (which require surplus land, income, livestock, and labor) or diversifying land use into woodlots (which demands surplus land). Households lacking these resources have limited access to newly important specific capacities (crop insurance, woodlots, and dry season planting areas) to effectively respond to risks of climatic variability and uncertainty and to adapt to climate change. The overall result is that an important segment of the smallholder farming population in Rwanda cannot effectively adapt livelihood and land-use portfolios to navigate structural changes and climatic changes.

The longer-term implications of the CIP in Rwanda bode poorly for pro-poor agricultural development. As this research demonstrates, negative synergies can arise when households with low generic capacity plant riskier commercial crops in lieu of traditional food security crops during periods of increasing climatic unpredictability. A series of climatic and other shocks can deplete a household of what little capitals it has, forcing adoption of livelihood activities that are not compatible with needed longer-term investments in livelihoods in ways that can create future opportunities for poverty reduction and increased wellbeing. To help promote sustainable adaptation for households across socio-economic groups, agricultural development policies must be mindful of the important role of local institutions in mitigating risk. Such an effort can be guided by attention to two questions about institutional change: 1) how does it differentially constrain and/or enable households’ traditional specific capacities? And 2) how does it differentially constrain and/or enable households to adapt livelihoods and land use to adopt new specific capacities? With looming climate change and visions of agriculture-led growth for SSA, both questions compel public leaders and private partners to face up to the potential for an economic growth paradox that pits the relative merits of higher productivity and incomes for already-advantaged households against the prospect of greater food insecurity among those at the lower end of the spectrum, for whom poverty and hunger continue to be ever-present realities.

This research addresses a deeper issue: whether the orientation of Green Revolution inspired agricultural development approaches towards economic growth precludes consideration of risk management as a policy component. These results suggest that large-scale intensification programs with externally prescribed quotas for planting export crops can exacerbate existing risks and introduce new risks in the form of crops ill-suited for the region or for particular parcels or farming systems. The negative impacts of cropping mandates are particularly evident during rainfall deviations and among households lacking capitals to invest in commercial crops (Clay, 2018b). These results further confirm the need for “localized, community-based efforts to increase local adaptive capacity” in highland East Africa (Thornton, Jones, Alagarswamy, Andresen, & Herrero, 2010). Absent such attention, crop productivity gains will come at the expense of disadvantaged individuals’ livelihoods. For Rwanda’s agricultural policy agenda, care should be taken to ensure that the most vulnerable groups do not slide further into poverty. Specifically, rather than applying the CIP indiscriminately across relatively arbitrary spatial jurisdictions that comprise administrative units, the intensification program should be designed to adaptively respond to spatial and temporal variations across the mountainous agro-ecological landscape. For households lacking the requisite generic capacities to adapt successfully, it is essential to consider options for easing the requirements of compulsory crop cultivation. This need for flexibility resonates across resource use systems, as a recent case from Botswana illustrates (King, Shinn, Yurco, Youngh, & Crews, 2018).

To broader work at the interface of climate and development, this paper contributes understanding of how uneven adaptive capacities can arise through interacting social-environmental processes over time (Watts, 1983). Development research and policy must actively consider such dynamics whether or not a study or program specifically targets climate adaptation. Optimistically, adaptation processes can embody the ‘potential to constitute or to contest authority, subjectivity and knowledge claims’ (Eriksen, Nightingale, & Eakin, 2015, p. 523) and active application of pathways approaches through participatory work and continuous reflection on management paradigms can empower individuals to take action to remake institutions (Wise et al., 2014). However, rigid institutions of environmental governance can also constrain livelihood adaptations (King, Shinn, Crews & Young, 2016). To visualize and implement transformative adaptation that actively reworks structural constraints and social inequalities (Bassett and Fogelman, 2013, Pelling, 2011), it is essential to understand how adaptive capacity is unevenly built during development transitions and within contexts of environmental change.

## Conflict of interest statement

The authors have no conflicts of interest to disclose. This article has not been submitted for publication elsewhere.

## Footnotes

1 The absent households may have resulted in an undersampling of certain household categories, likely poorer and one-adult households who were working throughout the days. We feel that this potential sampling bias does not affect the significance of the findings.
